# Supplementary material for: Embracing AI in academia: A mixed methods study of nursing students’ and educators’ perspectives on using ChatGPT
Source: PLoS One. 2025 Jul 17;20(7):e0327981. doi: 10.1371/journal.pone.0327981 (PMC12270142; doi:10.1371/journal.pone.0327981)
Supplement: S1 File — (DOCX) [file pone.0327981.s001.docx]

**Supplementary Table 1.** *Distribution of nurse educators according to their demographic characteristics (N= 40)*

| **Variable** | **No.** | **%** |
| --- | --- | --- |
| **Age** |  |  |
| <30 | 2 | 5.0% |
| 31-40 | 14 | 35.0% |
| 41-50 | 6 | 15.0% |
| 51-60 | 3 | 7.5% |
| Mean ± SD | 35.5 ± 7.6 | |
| **Nationality** |  |  |
| Saudi | 27 | 67.5% |
| Non-Saudi | 13 | 32.5% |
| **Position/Title** |  |  |
| Teaching Assistant | 3 | 7.5% |
| Lecturer | 5 | 12.5% |
| Assistant Professor | 24 | 60.0% |
| Professor | 3 | 7.5% |
| Associate Professor | 5 | 12.5% |
| **Years of Teaching Experience** |  |  |
| Less than 1-5 years | 7 | 17.5% |
| 6-10 years | 6 | 15.0% |
| 11-15 years | 10 | 25.0% |
| Over 15 years | 17 | 42.5% |
| Mean ± SD | \|  \| \| --- \|  \| 10.8 ± 4.2 \| \| --- \| |  |
| **Previous Experience with Chat GPT as AI Tools** |  |  |
| None | 4 | 10.0% |
| Moderate (used several times) | 28 | 70.0% |
| Extensive (regular use) | 8 | 20.0% |
| **Primary Reasons for Using ChatGPT in Nursing Academia** |  |  |
| Writing research papers or articles | 22 | 21.67% |
| Research assistance for curriculum development | 13 | 13.33% |
| Developing presentations | 13 | 12.78% |
| Student support and study assistance | 13 | 12.78% |
| Creating assignments | 11 | 11.11% |
| Translation for multilingual classrooms | 6 | 6.11% |
| Preparation of test questions | 5 | 5.00% |
| Editing | 2 | 2.22% |

**Supplementary Table 2.** *Distribution of nursing Students according to their demographic characteristics (N= 240).*

| **Variable** | **No.** | **%** |
| --- | --- | --- |
| **Age** |  |  |
| 19 | 36 | 15.0% |
| 20 | 73 | 30.4% |
| 21 | 121 | 50.4% |
| >21 | 10 | 4.2% |
| Mean ± SD | 20.5 ± 1.2 | |
| **Year of Study** |  |  |
| Second Year | 40 | 16.7% |
| Third year | 73 | 30.4% |
| Fourth year | 127 | 52.9% |
| **Previous Experience with Chat GPT as AI Tools** |  |  |
| None" | 35 | 14.6% |
| Minimal (used once or twice) | 67 | 27.9% |
| Moderate (used several times) | 84 | 35.0% |
| Extensive (regular use) | 54 | 22.5% |
| **Area of Chat GPT uses** |  |  |
| No | 35 | 14.6% |
| General information | 41 | 17.1% |
| Research | 39 | 16.3% |
| Assignment and plagiarism checker | 40 | 16.7% |
| Study of Nursing | 20 | 8.3% |
| Looking up fun facts | 20 | 8.3% |
| **Primary Reasons for Using ChatGPT in Nursing Studies*** |  |  |
| Study assistance | 174 | 72.5% |
| Assignment preparation | 132 | 55.0% |
| Research assistance | 108 | 45.0% |
| Translation | 105 | 43.8% |
| Presentation preparation | 95 | 39.6% |
| Test questions familiarization | 85 | 35.4% |
| Writing research papers | 70 | 29.2% |

- Multiple response

**Supplementary Table 3: ChatGPT in Academia - All Themes, Subthemes, and Shared/Different Points**

| **Themes (4)** | **Subthemes (22)** | **Sample Quotation (Nurse Educators (NE)** | **Sample Quotation Nurse Students (NS)** |
| --- | --- | --- | --- |
| **I. Uses of ChatGPT in Academia** | Writing Research Papers | *"I use ChatGPT for writing research papers to refine ideas and improve grammar."* | *"It helps me organize research papers by refining my writing and improving structure."* |
|  | Academic Support | *"I use it for sentence rephrasing, especially when I need to improve the clarity of my writing."* | *"ChatGPT helps me find reliable sources and summarize research topics for my assignments."* |
|  | Teaching and Learning | *"ChatGPT is a great tool for generating teaching ideas and simplifying complex medical information for my students."* | *"It helps me break down complex topics, especially in nursing techniques, into simpler steps."* |
|  | Other General Applications | *"I use ChatGPT for writing emails, translations, and sometimes to search for new ideas related to student assignments."* | ***"****It helps me gather information quickly and efficiently, sometimes also for refining my email and searching for funny idea."* |
| **II. Benefits & Positive Impact** | Writing Improvement and Summarizing Research | *"I used ChatGPT to improve the grammar and clarity of my writing, making my academic papers more polished."*  *"It helps me summarize research quickly, saving time during the writing process."* | *"ChatGPT helps me improve my writing, fix grammar mistakes, and make my work clearer."*  *"I use ChatGPT to condense long texts and structure my papers better."* |
|  | Collaboration and Support | *"ChatGPT has become a valuable collaborative tool, helping me refine my ideas and providing suggestions."*  *"It feels like having a colleague to bounce ideas off of.".* | *"ChatGPT helps me organize my research and improve my ideas by giving suggestions."*  *"ChatGPT provides useful input to refine my assignments."* |
|  | Time Saving and Efficiency | *"ChatGPT helps me save time by providing quick answers and summarizing long texts."*  *"It allows me to focus on deeper analysis instead of wasting time gathering basic information."* | *"ChatGPT saves me time by summarizing things quickly, so I can focus on understanding instead of searching."*  *"It speeds up my study process by summarizing key concepts."* |
|  | Efficient Research Search | *"ChatGPT helped me find related research studies much faster than traditional searches."*  *"It provides relevant references efficiently, saving my time.".* | *"ChatGPT helped me find sources quickly, saving a lot of time for my assignments."*  *"I use ChatGPT to identify articles I would have otherwise missed."* |
|  | Simplifying Complex Concepts | *"I used ChatGPT to simplify medical information into a more understandable format for different audiences."*  *"It simplifies clinical concepts, making teaching easier."* | *"ChatGPT helps me understand complex nursing concepts by breaking them down into simpler terms."*  *"ChatGPT breaks down difficult topics into smaller, easy-to-grasp ideas."* |
| **III. Weaknesses and Concerns** | Accuracy and Reliability of Information | *"I’ve noticed that ChatGPT doesn’t always provide the most accurate info, especially when it comes to research or literature.”* | *"Sometimes the answers from ChatGPT are incomplete or just flat-out wrong, which makes me double-check everything.”* |
| **Weaknesses** | Inaccessibility of References | *"The references ChatGPT provides aren’t always reliable or accessible, so I can’t always verify the information.”* | *"I often find that ChatGPT doesn’t provide proper sources for the information it gives me.”* |
|  | Lack of Ethical Guidelines | *"There’s a real gap in terms of guidelines on how much we should rely on ChatGPT. We need to know what’s okay and what’s not.”* | *"It’s important to know the limits—when should we use ChatGPT, and when is it better to go with traditional methods?" (NS)* |
|  |  |  |  |
| **Concerns** | Plagiarism and Ethical Issues | *"ChatGPT pulls from existing content, and I worry about unintentional copying.”* | *"ChatGPT might cause students to plagiarize if they don’t verify sources properly.”* |
|  | Over-reliance and Loss of Skills | *"I worry that if students rely too much on ChatGPT, they’ll lose their ability to think critically and do their own research.”* | *"Using ChatGPT too often could make students lazy and stop them from doing their own thinking or research.”* |
|  | Impact on Research Integrity | *"Using ChatGPT could hurt the integrity of research, especially if students start depending on AI-generated content instead of doing their own work.”* | *"ChatGPT might encourage lazy habits, where students copy information without thinking critically or checking sources.”* |
|  | Ethical Concerns in Data Security | *"There’s concern about privacy when using ChatGPT, especially when it comes to sensitive data. It could expose information we don’t want to share.”* | *"ChatGPT might expose our academic data if we’re not careful, and there’s a risk of data breaches.”* |
| **IV. Suggested Improvements**  **For future use** | Valid Referencing, Source Transparency, and Accuracy | *I would really appreciate it if ChatGPT could provide clear references and mention where the information comes from, so I can be sure it’s reliable.* | *I think it would help if ChatGPT could link to scientific journals or websites to make the research more credible.* |
|  | Clear Ethical Guidelines and Responsible Usage | *It’s crucial that ChatGPT has clear rules for when it should and shouldn’t be used, especially to maintain academic integrity.* | *It would be helpful to have some guidelines on how to use ChatGPT ethically, so we don’t accidentally misuse it.* |
|  | Specialized and In-Depth Information | *It would be really helpful if ChatGPT could provide more specialized content that’s deeper and more relevant to specific nursing fields.* | *I wish ChatGPT could give more detailed and specific information related to nursing, especially for my research projects.* |
|  | Training, Workshops, and Orientation | *I think there should be workshops or training sessions so that we can get the most out of ChatGPT while understanding its limitations.* | *It would be great if ChatGPT had tutorials or workshops that teach us how to use it effectively for our studies.* |
|  | Academic Tools and Features | *Having features like academic flashcards to help students review key concepts quickly would be really useful.* | *It would be helpful if ChatGPT could generate flashcards or summaries to help me revise for exams.* |
|  | Ease of Use and Efficiency | *ChatGPT needs to be more intuitive and save time, especially when it comes to organizing resources or structuring my lectures.* | *It would be awesome if ChatGPT was easier to use, helping me save time when I’m looking for answers or doing my assignments.* |
