# Supplementary material for: Embracing AI in academia: A mixed methods study of nursing students’ and educators’ perspectives on using ChatGPT
Source: PLoS One. 2025 Jul 17;20(7):e0327981. doi: 10.1371/journal.pone.0327981 (PMC12270142; doi:10.1371/journal.pone.0327981)
Supplement: Attachment — Submitted filename: Comments.docx [file pone.0327981.s003.docx]

This study provides valuable insights into the role of AI in nursing education. Addressing the concerns outlined below more explicitly will enhance the study’s overall impact.

**Thematic Analysis Rigor**

1. The qualitative analysis mentions Braun and Clarke’s framework but does not specify how intercoder reliability was ensured. Were multiple researchers involved in coding? If so, how were disagreements resolved? Providing more details would enhance the trustworthiness of the findings.

**Limitations**

2. While the study acknowledges certain limitations, it should discuss how these might impact the findings and interpretations. For instance, the sample includes students and educators from a single institution. How might this affect the generalizability of the results? Consider discussing whether similar findings could be expected in other cultural or educational settings.

**Detailed Discussion on Ethical Concerns**

3. The ethical concerns regarding ChatGPT’s use (plagiarism, over-reliance, and privacy issues) are well-identified. However, the study would benefit from a deeper discussion on potential solutions. Could existing ethical frameworks in nursing education provide guidance?

Sincerely

Moustaq Karim Khan Rony
