# Supplementary material for: Embracing AI in academia: A mixed methods study of nursing students’ and educators’ perspectives on using ChatGPT
Source: PLoS One. 2025 Jul 17;20(7):e0327981. doi: 10.1371/journal.pone.0327981 (PMC12270142; doi:10.1371/journal.pone.0327981)
Supplement: Attachment — Submitted filename: Response_Letter_to_Reviewer_Comments.docx [file pone.0327981.s006.docx]

# Response Letter to Editor’s Comments

## PONE-D-24-60162

Embracing AI in Academia: A Mixed Methods Study of Nursing Students' and Educators' Perspectives on Using ChatGPT

To:
Dr. Florie Lacorte Canales
PLOS ONE Editorial Team

Dear Dr. Lacorte Canales,

Thank you for your detailed feedback and the opportunity to revise our manuscript. We have carefully addressed all the comments to ensure compliance with PLOS ONE’s submission guidelines. Below is our point-by-point response:

### 1. Completion of the Human Participants Research Checklist

We have completed all relevant sections of the PLOS ONE Human Participants Research Checklist. The completed checklist has been attached to this resubmission as a separate file under the file type “Other.”

### 2. Ethics Committee Approval

We have updated the Methods section and the “Ethics Statement” field in the submission form with the following text:

### The study was approved by both the CONJ Research Committee and the King Abdullah International Medical Research Center’s (KAIMRC) Institutional Review Board under approval number NRJ24/034/8. Prior to participation, students and educators were fully informed about the study’s purpose, including their right to refuse or withdraw at any time without any consequences. Written informed consent was obtained from all participants, and strict measures were implemented to ensure data privacy and confidentiality. Anonymity was maintained by using pseudonyms or participant numbers in the reporting of interview data.

### 3. Details Regarding Participant Consent

We have clarified the details of participant consent in the manuscript and the online submission form:

- Type of Consent: Written informed consent was obtained from all participants.

- Documentation: Signed consent forms were collected before participation.

- Minors: No minors were included in this study.

- Waiver of Consent: A waiver of consent was not applicable.

## Attachments

The following documents are attached to this resubmission:

1. Completed Human Participants Research Checklist

2. Ethics Committee IRB Approval Letter

3. Informed Consent Template

We HOPE that these revisions and the provided documentation align with PLOS ONE’s requirements. If any further clarification or adjustments are needed, please do not hesitate to contact us.

Thank you for your time and consideration.

Kind regards,
Prof. Ebtsam Aly Abou Hashish
